# Proteomics analysis of tissue samples from patients with squamous cell carcinoma of the penis and positive to human papillomavirus

**DOI:** 10.1590/S1677-5538.IBJU.2014.0051

**Published:** 2015

**Authors:** Leandro Koifman, Paulo Ornellas, Antonio Augusto Ornellas, Denise de Abreu Pereira, Benedeta Russolina Zingali, Silvia Maria Baeta Cavalcanti, Larissa Alves Afonso, Vanessa Sandim, Gilda Alves

**Affiliations:** 1 Serviço de Urologia, Hospital Municipal Souza Aguiar, Rio de Janeiro, Rio de Janeiro, Brasil; 2 Serviço de Urologia, Hospital Mário Kröeff, Rio de Janeiro, Rio de Janeiro, Brasil; 3 Serviço de Hematologia, Instituto Nacional de Câncer - Laboratório de Genética Aplicada, Rio de Janeiro, Rio de Janeiro, Brasil; 4 Programa de Pós-Graduação em Ciências Médicas (PGCM), Universidade Estadual do Rio de Janeiro, Rio de Janeiro, Brasil; 5 Departmento de Urologia, Instituto Nacional de Câncer, Rio de Janeiro, Brasil; 6 Instituto Nacional de Câncer - Programa de Carcinogênese Molecular, Coordenação Geral de Ensino e Pesquisa, Rio de Janeiro, Brasil; 7 Universidade Federal do Rio de Janeiro - Instituto de Bioquímica Médica, Unidade de Espectrometria de Massas e Proteômica, Instituto Nacional de Biologia Estrutural e Bioimagem (INBEB), Rio de Janeiro, Brasil; Universidade Federal do Rio de Janeiro - Instituto de Bioquímica Médica, Unidade de Espectrometria de Massas e Proteômica, Instituto Nacional de Biologia Estrutural e Bioimagem (INBEB), Rio de Janeiro, Brasil; 8 Universidade Federal Fluminense - Laboratório de Diagnóstico Virológico, Departamento de Microbiologia e Parasitologia, Instituto Biomédico, Rio de Janeiro, Brasil

**Keywords:** Carcinoma, Squamous Cell, Penis, Human papillomavirus

## Abstract

**Purpose:**

The aim of this study was to identify possible protein biomarkers and/or candidates for therapeutic targets in tissues of patients with SCCP, infected by HPV, applying one dimensional electrophoresis (1DE), followed by direct mass spectrometry (MS) analysis.

**Materials and Methods:**

Tissues from 10 HPV positive patients with SCCP and from 10 patients with HPV negative non-tumorous penile foreskins were analyzed applying 1D electrophoresis, followed by analysis with direct mass spectrometry (MS).

**Results:**

Sixty-three different proteins were identified in the first group and 50 in the second group. Recognition was possible for 28 proteins exclusively detected in Group 1 and 21 proteins presented only in Group 2.

**Conclusion:**

Some proteins in the first group are directly involved in the development of other types of cancer, and therefore, suitable for analysis. Complement C3 protein is a strong candidate for evaluating SCCP patients.

## INTRODUCTION

Cancer of the penis is a rare neoplasm with a high incidence in developing countries. This fact clearly indicates the disease's association with local economic conditions (1). Penile cancer has a low overall incidence, representing approximately 0.4% of malignancies in the Unites States. In Brazil, despite the high incidence in some regions, this disease accounts for about 2.1% of malignancies (2, 3). A recent Brazilian epidemiologic study on penile carcinoma revealed the profile of these patients (4).

The etiology of penile cancer has not been fully elucidated. However, its incidence varies according to the practice of circumcision, personal hygiene, presence of phimosis, human papillomavirus (HPV) infection, and tobacco use (5–9). The mechanism of tumor induction and promotion related to HPV infection is not completely understood. It is believed that the incorporation of viral DNA to the human genome leads to hyper-expression of viral genes E6 and E7 and inactivates the host cell's tumor suppressor gene products p53 and pRb (10).

The presence and extent of inguinal metastases are the most important prognostic factor related to the survival of patients with penile carcinoma. At the time of its initial presentation, 50% of patients with SCCP have inguinal lymphadenopathy; however, only half of these actually show metastatic lymph node involvement. Furthermore, 20% of patients with clinically negative inguinal lymph nodes have micro-metastases that will only be diagnosed by histopathologic examination of surgical specimens obtained from lymphadenectomy, a procedure associated with a significant morbidity (1, 4). Therefore, SCCP remains a challenge for the urologist, because there is no consensus for an appropriate therapy for all forms of disease presentation. The possibility of using reliable biomarkers to predict disease prognosis and to establish procedures less aggressive for patients at low risk for metastasis becomes necessary. In this sense, the development of more accurate molecular diagnostic methods and prognostic value tumor markers is essential.

Proteomics is the large-scale identification of proteins. Proteomics technologies are currently under development and several methodological approaches can be applied depending on the objectives. The great advantage of proteomics over genomics or transcriptomics studies is that the real functional molecules of the cell are being studied. Therefore, in this study, the aim was to identify possible protein biomarkers and/ or candidates for therapeutic targets in tissues of patients with SCCP, infected by HPV, applying one dimensional electrophoresis (1DE), followed by direct mass spectrometry (MS) analysis.

## MATERIALS AND METHODS

### Patients and controls

Between January 2009 and December 2011, 20 patients treated at three health institutions in the state of Rio de Janeiro were recruited and divided into two groups for prospective tissue proteomic analysis. Group 1 was composed of 10 patients with positive HPV malignant SCCP treated at the Brazilian National Cancer Institute (INCA) and Mario Kröeff Hospital. Group 2 (control group) was composed of 10 patients with HPV negative non-tumorous penile foreskins collected at Santa Veronica Hospital after circumcision procedures. HPV typing was performed as previously published (11, 12) and reported (13).

Pathological material was reviewed in both groups and all tumors were histologically classified based on Broders’ system. Only two pathologists were responsible for reviewing the specimens. The clinical and pathological staging for Group 1 was done according to the 2002 TNM classification system. Patients’ treatment varied according to primary tumor presentation. The distribution of T and N categories is shown in Table-1. The criterion for performing a radical inguinal lymphadenectomy (RIL) in all cases was the stage, grade and/or presence of lymphovascular invasion. Two patients with stage T1 underwent lymphadenectomy. The first presented unilateral inguinal lymphadenopathy and the second presented grade 2 tumor. In our services, we only do not perform RIL in patients with stage T1N0M0 grade 1 tumor without lymphovascular invasion ages ranged from 38 to 90 years (mean, 63.56) for Group 1 and 23 to 83 years (mean, 60) for Group 2. The pathological variables studied were histological type, grade of tumor differentiation, corpus spongiosum and/or infiltration of the corpora cavernosa, urethral in-filtration, and inguinal lymph nodes involvement. Patient recruitment did not take into account any criteria of poor prognosis, and the tissue specimens were randomly selected respecting the number of pre-established patients for the study. All patients involved in the current study gave their informed consent. This study was approved by the Brazilian National Cancer Institute Ethical Board (registrations # 38/05 and 67/07). Because this was a pilot study and unprecedented in literature the number of patients was pre-established in both groups in the design of work, aiming preliminary results for further investigation. The only exclusion criterion was positivity for HPV in the control group. Our study aimed qualitative detection of proteins in the 2 groups not being our objective to quantify the identified proteins. All tests in tumor samples from patients revealed the presence of HPV. Because of the rarity of HPV-negative patients, a second study with HPV-negative patients will be necessary.

**Table 1:** Histopathologic findings, pathologic staging and treatment option for patients from group 1.

Pts	Histology	Grade	Stage TNM	HPV type	Surgery
1	Squamous cell carcinoma	G2	T4N2Mx	MY-/16+	Total Amputation + Bilateral RIL
2	Squamous cell carcinoma	G1	T2N3Mx	MY-/18+	Partial Amputation + Bilateral RIL
3	Squamous cell carcinoma	G2	T2N0Mx	16+;45+	Partial Amputation + Bilateral RIL
4	Squamous cell carcinoma	G2	T2N0Mx	MY-/18+	Partial Amputation + Bilateral RIL
5	Squamous cell carcinoma	G1	T1N1Mx	MY-/45+	Partial Amputation + Bilateral RIL
6	Squamous cell carcinoma	G2	T1N0Mx	45+	Partial Amputation + Bilateral RIL
7	Squamous cell carcinoma	G1	T2N0Mx	MY-/16+ 45+	Partial Amputation + Bilateral RIL
8	Squamous cell carcinoma	G2	T2N1Mx	MY+/45+	Partial Amputation + Bilateral RIL
9	Squamous cell carcinoma	G2	T2N1Mx	MY+/16+	Partial Amputation + Bilateral RIL
10	Squamous cell carcinoma	G2	T2N0Mx	MY-/45+	Partial Amputation + Bilateral RIL

RIL = Radical Inguinal Lymphadenectomy; MY-MY09/11 Consensus Primers

### Tissue protein extraction and quantification

Tissues were macerated in 200μL of lysis buffer (7 M urea, 2 M Thiourea, 4% CHAPS and 1% DTT) with the addition of 0.2-mM PMSF. This mixture was stirred for 1 hour at room temperature and then centrifuged at 14.000g for 15 minutes. The supernatant was collected and stored at −80°C (14) until experimentation.

The protein extracts were quantified by 2D Quant Kit (GE Healthcare, Cat #. 80-6483-56), according to the manufacturer's instructions. Measurement was performed at 650 nm in Elisa Spectra Max 190 device from Molecular Devices. The analysis of quantification was performed by the program SOFT® Pro 4.3 max, Life Sciences Edition.

### Gel 1D

After quantification, two protein pools were formed with 10 SCCP tissues and with 10 control tissues, separately. Each pool contained 3.3μg of proteins from each sample, a total of 33μg. The SCCP and control pools were applied on a 12% SDS-PAGE gel. Proteins were separated in Tris-Glycine buffer (25-mM Tris and 250-mM Glycine pH 8,3) and 0.1% SDS at 80 V and 50 mA (15). The proteins were visualized with Coomassie blue G-250. The gels were scanned on Image ScannerTM (GE Healthcare) using the program Labscan™ (GE Healthcare) for protein lanes reading.

### Mass spectrometry analysis

The lanes were fractioned in approximately 2-5 mm slices. The bands in the slices were destained in a solution of 25-mM ammonium bicarbonate (NH_4_HCO_3_) pH 8.8/50% and acetonitrile (ACN) overnight on a shaker, at room temperature. To reduce proteins, the gel was incubated with 10mM DTT in 25-mM NH_4_HCO_3_ at 56°C for 1 hour. The supernatant was discarded and the gel was washed in a solution of 25-mM NH_4_HCO_3_ twice. After protein disulfide bonds were reduced, cysteines were alkylated with iodoacetamide 55 mM for 45 minutes at room temperature in the dark. The supernatant was discarded and the gel was washed with 25-mM NH_4_HCO_3_ solution in 50% ACN. The supernatant was removed again and gel slices were dehydrated with 100% ACN for 5 minutes and posteriorly in a vacuum centrifuge. Proteins were digested with trypsin (Promega) 10ng/μL dilution, overnight, at 37°C. After digestion with trypsin, peptides were extracted from gels by adding a solution containing 0.1% formic acid/50% ACN for 30 minutes. This solution was transferred to another tube and the procedure was repeated twice. The samples were completely dried in a vacuum centrifuge. The pellets were resuspended in water and purified through Ziptip Perfect Pure C18 (Eppendorf, cat # 0030.008.405) and then dried in a vacuum centrifuge.

For mass spectrometry analysis, the peptides were resuspended in 20μL of acetonitrile 3% and acid formic 0.1% solution. The peptides were analyzed by mass spectrometer ESI-Q/TOF Micro (Waters) linked to a nanoACQUITYUPLC® (Waters). The peptides was loaded on symmetric C18 trap column (Waters) followed by fraction in a nanoEase BEH 130 C18 100 mm × 100μm column (Waters) at a flow rate of 0.5μL/min and eluted with a linear acetonitrile gradient (from 10 to 50%) of 0.1% formic acid. Spectrometer analysis was performed on positive mode. Acquisition parameters on mass spectrometer was: cone voltage 30 V, capillary voltage 3500 V, source temperature 80°C, scanning a mass-to-charge ratio (m/z) MS mode 400-2000 and MS/MS mode 50-2000. The three ions with more intensity with charge states of +2, +3, or +4 were selected for MS/MS fragmentation. The reference ion used was the monocharged ion m/z 588.8692 of phosphoric acid. The data acquisition was performed by MassLynx 4.0 software (Micromass/Waters) and the process data by proteinLynx Global Service (PLGS 2.4, Waters).

Proteins were identified by correlation of tandem mass spectra to the NCBInr proteins database, using Mascot online (Matrix Science, London, UK - http://www.matrixscience.com/cgi/se-arch_form.pl?FORMVER=2&SEARCH=MIS) with restricted taxonomy Homo sapiens. The NCBI (National Center for Biotechnology Information) protein database is an on line collection of sequences from several sources, including translations from annotated coding regions in GenBanK, RefSeq and TPA, as well as records from Swiss-Prot, PIR, PRF, and PDB; “nr” refers to non-redundant protein sequences. The NCBI is a division of the National Library of Medicine (NLM) at the National Institutes of Health (NIH), USA. The parameters were as follows: MS and MS/MS tolerance of 0.1 Da, tryptic specificity allowing for one missed cleavage, fixed modification of carbamidomethylation of cysteine residues, and variable modification of oxidation of methionine, phosphorylation of tyrosine, serine and threonine residues and propionamide. Positive protein identification was accepted with at least two peptides with a Mascot peptide score ≥35.

## RESULTS

The pathological features of the primary tumor and inguinal lymph nodes, the type of treatment instituted, and the HPV type (13) for each patient from Group 1 are described in Table-1.

**Figure 1:**
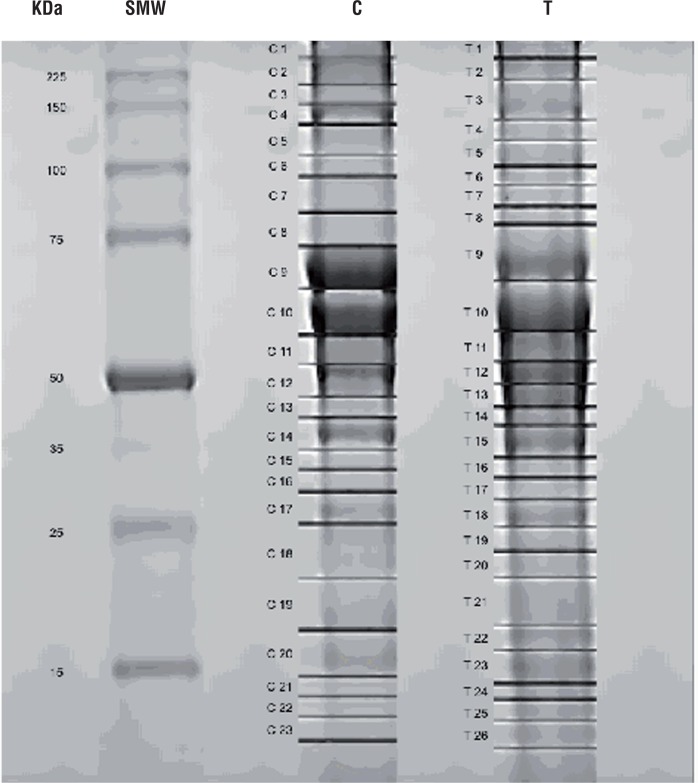
1DE analysis of tissue samples from SCCP HPV patients and control group. Each pool contained 3.3µg of proteins from each sample, a total of 33µg. The SCCP and control pools were applied on a 12% SDS-PAGE gel. The gel was stained with Coomassie blue G. The markers and numbers in gel represent the sections that were excised for mass spectrometry analysis.

The protein extracts obtained from Groups 1 and 2 were separated by electrophoresis in a 12% SDS-PAGE gel. The protein bands of each group were identified and compared for differences. Twenty-six protein spots from Group 1 and 21 from Group 2 were identified, sliced out from the gel and analyzed through mass spectrometry (Figure-1). Sixty-three different proteins were identified in Group 1 and 50 in Group 2. After a comparative analysis of both groups, it was possible to recognize 28 proteins exclusively detected in Group 1 and 21 proteins presented only in Group 2 (Tables 2 and 3).

**T**=penile cancer SCCP; **C**=control group; **SMW**=Standard Molecular Weight

**Table 2:** Proteins identified in pool of patients with SCCP (Group 1)

Gel Slice	NCBI ID	Proteins	Score	Representative Peptides	KEGG	Function	Cell Compartment
T1	gi|179212	Na+ K+ ATPase alpha subunit	41	R.SPDFTNENPLETR.N	hsa:476	ATP biosynthesis	Cell membrane
T2;3	gi|189036	Nonmuscle myosin heavy chain (Myosin 9)	78	K.ALELDSNLYR.I K.HSQAVEELAEQLEQTKR.V	hsa:4627	Cell communication	Cytosol
T2	gi|1296662	Plectin	74	R.SQVEEELFSVR.V K.VLALPEPSPAAPTLR.S	hsa:5339	Apoptosis	Cytosol, cytoskeletal
T2	gi|93141049	Collagen alpha-1 (XII) chain short isoform precursor	60	K.ALALGALQNIR.Y R.WYSPVDGTRPSESIVVPGNTR.M	hsa:1303	Cell adhesion	Extracellular matrix
T5	gi|4507677	Endoplasmin	44	K.SILFVPTSAPR.G	hsa:7184	Proteins processing	Cytosol
T6	gi|223170	Fibrinogen gamma	58	K.EGFGHLSPTGTTEFWLGNEK.I K.MLEEIMKYEASILTHDSSIR.Y K.AIQLTYNPDESSKPNMIDAATLK.S	hsa:2266	Blood coagulation	Secreted
T9	gi|5729877	Heat Shock cognate 71-kDa protein isoform 1	41	K.DAGTIAGLNVLR.I(U)	hsa:3312	Stress response, transcription regulation	Cytosol, nucleus, cell surface
T11	gi|2982019	Chain B, Fab Fragment of engineered Human Monoclonal Antibody A5b7	47	K.GPSVFPLAPCSR.S/ R.STSESTAALGCLVK.D/ E.VQLLESGGGLVQPGGSLR.L	Unknown	Cell defense	Secreted
T11;12	gi|178375	Aldehyde dehydrogenase	44	K.LPEWAADEPVEK.T/ R.SLEEAIQFINQR.E	hsa:218	Glycolysis, metabolism of amino acids and xenobiotics	Cytosol
T13	gi|4503571	Alpha-enolase isoform 1		R.GNPTVEVDLFTSK.G/ R.YISPDQLADLYK.S/ K.WIGMDVAASEFFR.S + Oxidation (M) / K.VNQIGSVTESLQACK.L / K.YNQLLRIEEELGSK.A/	hsa:2023	Glicolysis; gliconeogenese	Cytosol
			90	K.LAMQEFMILPVGAANFR.E + 2 Oxidation (M) /R.EIFDSRGNPTVEVDLFTSK.G/ K.DATNVGDEGGFAPNILENKEGLELLK.T			
T13	gi|1710248	Protein disulfide isomerase-related protein 5	55	R.TGEAIVDAALSALR.Q/ K.LAAVDATVNQVLASR.Y	hsa:10130	Proteins processing	Endoplasmatic reticulum
T13	gi|31170	Chain A, Crystal Structure Of Human Beta Enolase Enob	44	K.VNQIGSVTESIQACK.L	hsa:2027	Glicolysis	Cytosol, fosfopiruvat hydratase complex
T13	gi|40068518	6-phosphogluconate dehydrogenase, decarboxylating	42	K.IISYAQGFMLLR.Q + Oxidation (M) / K.GILFVGSGVSGGEEGAR.Y	hsa:5226	Pentose pathways	Cytosol
T14	gi|4505763	Phosphoglycerate kinase 1	39	K.ITLPVDFVTADKFDENAK.T	hsa:5230	Glicolysis	Cytosol
T14	gi|306882	Haptoglobin precursor	36	K.VTSIQDWVQK.T/ K.SPVGVQPILNEHTFCAGMSK.Y + Oxidation (M)	hsa:3240	Defense	Secreted
T15	gi|35222	70-kDa heat shock protein	43	R.TTPSYVAFTDTER.L (U)	hsa:3312	Regulation of cell cycle; cellular membrane organization	Cytosol; plasma membrane
T16	gi|63252913	Macrophage-capping protein	44	R.QAALQVAEGFISR.M	hsa:822	Actin filament organization	Cytosol
T17	gi|5174391	Alcohol dehydrogenase [NADP+]	44	K.GLVQALGLSNFNSR.Q/ R.GLEVTAYSPLGSSDR.A	hsa:10327	Glicolysis, glicerolipids metabolism	Cytosol
T17	gi|31397	Fibronectin precursor	40	R.VPGTSTSATLTGLTR.G	hsa:2335	Angiogenesis, cell adhesion, platelet activation and degranulation	Secreted, extracellular matrix
T18	gi|31645	Glyceraldehyde- 3-phosphate dehydrogenase	46	R.GALQNIIPASTGAAK.A/ R.VPTANVSVVDLTCR.L/ K.LISWYDNEFGYSNR.V/ K.LTGMAFRVPTANVSVVDLTCR.L + Oxidation (M) / K.IKWGDAGAEYVVESTGVFTTMEK.A + Oxidation (M) / K.VIHDNFGIVEGLMTTVHAITATQK.T + Oxidation (M)	hsa:2597	Glycolysis	Cytosol, plasma membrane
T19;20	gi|809185	Chain A, The Effect Of Metal Binding On The Structure Of Annexin V And Implications For Membrane Binding	52	RSEIDLFNIRK/ KGLGTDEESILTLLTSRS/ KWGTDEEKFITIFGTRS/ RGTVTDFPGFDERADAETLRK	hsa:308	Blood coagulation	Cytosol
T19	gi|2906146	Malate dehydrogenase precursor	46	K.IFGVTTLDIVR.A/ K.VDFPQDQLTALTGR.I	hsa:4191	Citric acid cycle	Mitochondria
T19	gi|4826643	Annexin A3	37	K.MLISILTER.S +Oxidation (M)/ K.GAGTNEDALIEILTTR.T	hsa:306	Defense response	Phagocytic vesicle
T19	gi|4929769	Glyoxalase domain-containing protein 4 (CGI-150 protein)	36	K.ILTPLVSLDTPGK.A(U)	hsa:51031	Unknown	Mitochondria
T20	gi|4502599	Carbonyl reductase [NADPH] 1	46	R.LFSGDVVLTAR.D/ R.VVNVSSIMSVR.A/ R.GQAAVQQLQAEGLSPR.F/ K.VADPTPFHIQAEVTMK.T + Oxidation	hsa:873	Lipid metabolism -arachidonic acid	Cytosol
T23	gi|9844110	cAMP-specific phosphodiesterase 4D	42	K.LSPVISPR.N	hsa:5144	Smooth muscle contraction; regulation of receptor activity	Cytosol
T24	gi|2204207	Glutathione S-transferase	75	M.PPYTVVYFPVR.G -.MPPYTVVYFPVR.G + Oxidation (M) M.PPYTVVYFPVRGR.C K.EEVVTVETWQEGSLK.A K.FQDGDLTLYQSNTILR.H K.ALPGQLKPFETLLSQNQGGK.T K.YISLIYTNYEAGKDDYVK.A	hsa:2940	Amino acids metabolism	Cytosol
T26	gi|181250	Cyclophilin	20	K.TVDNFVALATGEK.G	hsa:5480	Signal transduction	Cytosol

**Table 3:** Proteins identified in pool of patients with non-tumor tissue (Group 2).

Gel Slice	NCBI ID	Proteins	Score	Representative Peptides	KEGG	Function	Cell Compartment
C1	gi|78101267	Chain A, Human Complement Component C3	57	K.TIYTPGSTVLYR.I R.IPIEDGSGEVVLSR.K R.LVAYYTLIGASGQR.E	hsa:718	Inflammatory response; innate immunity; lipid metabolism	Secreted
C2	gi|28243	Filamin A	49	R.IANLQTDLSDGLR.L R.SAGQGEVLVYVEDPAGHQEEAK.V K.LDVQFSGLTK.G K.SPFSVAVSPSLDLSK.I R.EGPYSISVLYGDEEVPR.S R.FGGEHVPNSPFQVTALAGDQPSVQPPLR.S	hsa:2316	Actin coupler	Cytosol
C3	gi|2104553	Myosin heavy chain (MHY11)(5′partial)	59	K.HAQAVEELTEQLEQFKR.A R.ALEEALEAKEELER.T K.IAQLEEQVEQEAREK.Q	hsa:4629	Vascular muscle contraction; cell communication	Extracellular matrix
C7	gi|34228	Prelamin-A/C isoform 1 precursor; lamin A protein; progerin	56	R.VAVEEVDEEGKFVR.L	hsa:4000	Intermediate filament	Nucleus
C7	gi|386758	GRP78 (Glucose-regulated protein 78) precursor	45	R.ITPSYVAFTPEGER.L R.IINEPTAAAIAYGLDKR.E	hsa:3309	Anti-apoptosis	Endoplasmic reticulum
C7	gi|23200154	Chain A, NMR Structures Of The C-Terminal Globular Domain Of Human Lamin AC	43	R.VAVEEVDEEGKFVR.L	hsa:4000	Structural molecule activity	Nucleus
C8	gi|762885	Plakoglobin	72	K.SAIVHLINYQDDAELATR.A R.ALMGSPQLVAAVVR.T Oxidation (M) R.LVQNCLWTLR.N R.NEGTATYAAAVLFR.I	hsa:3728	Cell adhesion, cell migration	Cytosol; plasmatic membrane
C8	gi|642534	Lumican	42	K.SLEYLDLSFNQIAR.L	hsa:4060	Collagen fibrils organization	Extracellular matrix
C8	gi|110590597	Chain A, Apo-Human Serum Transferrin (Non-Glycosylated)	36	K.FYLGYEYVTAIR.N	hsa:7018	Mineral absorption	Secreted
C10	gi|35505	Pyruvate kinase	39	R.TATESFASDPILYRPVAVALDTKGPEIR.T R.EAEAAIYHLQLFEELRR.L	hsa:5315	Glycolysis	Cytosol; plasmatic membrane
C11;13; 15; 16	gi|3411130	Mutant Desmin	48	R.FLEQQNAALAAEVNR.L	hsa:1674	Cytoskeleton structural protein activity	Intermediate filament C
C13	gi|340219	Vimentin	60	K.ILLAELEQLK.G K.ILLAELEQLKGQGK.S K.LQEEMLQREEAENTLQSFR.Q Oxidation (M) R.KVESLQEEIAFLK.K R.QVQSLTCEVDALKGTNESLER.Q R.EYQDLLNVK.M K.MALDIEIATYR.K Oxidation (M) R.ISLPLPNFSSLNLR.E	hsa:7431	Apoptosis; cell mobility	Cytosol
C13	gi|704416	Elongation factor Tu	45	K.LLDAVDTYIPVPAR.D	hsa:7284	Oxidative phosphorylation	Mitochondria
C15	gi|34234	Laminin-binding protein	75	R.AIVAIENPADVSVISSR.N R.FTPGTFTNQIQAAFREPR.L	hsa:3921	Ribosome	Cytosol
C16	gi|47519616	Tropomyosin beta chain isoform 2	73	R.IQLVEEELDR.A R.IQLVEEELDRAQER.L R.LATALQKLEEAEK.A	hsa:7169	Muscle contraction	Cytosol
C17	gi|31645	Glyceraldehyde-3-phosphate dehydrogenase	72	K.VIHDNFGIVEGLMTTVHAITATQK.T Oxidation (M) R.DGRGALQNIIPASTGAAK.A R.GALQNIIPASTGAAK.A R.VPTANVSVVDLTCR.L K.LISWYDNEFGYSNR.V	hsa:2597	Glycolysis	Cytosol; plasmatic membrane
C19	gi|4505773	Prohibitin	91	R.IFTSIGEDYDER.V R.FDAGELITQR.E	hsa:5245	DNA synthesis	Mitochondrial membrane
C19	gi|66473265	Beta globin chain	50	K.VNVDEVGGEALGR.L R.LLVVYPWTKR.F	hsa:5245	Oxygen transport	Hemoglobin
C21	gi|494066	Chain A, Three- Dimensional Structure Of Class Pi Glutathione S-Transferase From Human Placenta In Complex With S-Hexylglutathione At2.8 Angstroms Resolution	36	.PPYTVVYFPVRGR.C K.FQDGDLTLYQSNTILR.H	hsa:2940	Amino acids metabolism	Cytosol

## DISCUSSION

A large number of proteins were identified in both Groups 1 and 2. Some of these proteins found in Group 1 are also directly involved in the development of other types of cancers and therefore, suitable for analysis.

The major stress-inducible heat shock protein, Hsp70, that is a chaperone protein abundantly and preferentially expressed in tumors, was detected in Group 1. Owing to the ability of Hsp70 to protect cells from a wide range of apoptotic and necrotic stimuli, it has been assumed that Hsp70 may confer survival advantage to tumor cell lines. Nylandsted et al. (16) demonstrated that the depletion of Hsp70 by an adenovirus expressing antisense Hsp70 resulted in a massive cell death of tumorigenic cell lines of breast, colon, prostate, and liver. The authors advocate that Hsp70 is a prerequisite for the survival of human cancer cells. Similarly, Aghdassi et al. (17) demonstrated that the depletion of Hsp70 by short interfering RNA treatment induced apoptosis in pancreatic adenocarcinoma.

Plectin is a cytolinker protein of the plakin family. Plakins connect intermediate filaments to desmosomes and hemidesmosomes, stabilize cells mechanically, regulate cytoskeleton dynamics, and serve as a scaffolding platform for signaling molecules. Niwa et al. (18) reported that Plectin misexpression leads to displacement of the centrosome, therefore contributing to genomic instability and cancer development. Nevertheless, plectin is not expressed by most normal tissues, with the exception of the skin and genitourinary tract. Interestingly, we have detected plectin solely in Group 1. Complement plays a central part of the innate immune system, providing a highly effective means for destruction of invading microorganisms: clearance of immune complexes; and elimination of dead, apoptotic, and tumor cells. During the evolution of a cancer cell, neo-antigens are produced. These elements distinguish cancer cells from their normal counterparts and may well be recognized by the immune system, eliminating many or most tumors (19, 20). Although most in vivo observations support that many cancers activate the autologous complement system, it is also well-known that the efficiency of complement-mediated tumor cytotoxicity is hampered by various protective mechanisms (21). In this work, human complement C3 was detected only in Group 2. A possible explanation for these findings lies on the theory that patients with malignancies have a poorer immune response. Our result corroborates the study of Ornellas et al. (22), in which the authors have demonstrated that human complement fragments C3 and C4A/B were downregulated in plasma of patients with SCCP. In the present series, all patients from Group 1 were HPV positive and this could explain the absence of complement C3 because viral proteins counteract the immune response (23).

Enolase is a key glycolytic enzyme that has been used as a diagnostic marker to identify human lung cancers (24). Higher α-enolase plasma levels were also identified in patients with renal cell carcinoma (25). In cancer cells, enolase is overexpressed and localizes on their surface, where it acts as a key protein in tumor metastasis, promoting cellular metabolism in anaerobic conditions and driving tumor invasion through plasminogen activation and extracellular matrix degradation. It also displays a characteristic pattern of acetylation, methylation, and phosphorylation that regulates protein functions and immunogenicity. In the present study, alfa and beta enolase isoforms were identified exclusively in Group 1. This finding may suggest that in the future, enolase can be used as a possible clinical biomarker. Nevertheless, further studies are needed to corroborate these findings and to determine the usefulness of this protein in clinical scope.

Prohibitin is a potential tumor suppressor, which was originally identified because of its anti-proliferative activities. The human prohibitin gene was identified and cloned in 1991, as a result of a search for potential tumor suppressors, on the basis of its anti-proliferative activities (26). Furthermore, prohibitin is capable of inhibiting cell proliferation by repressing the transcriptional activity mediated by E2F which regulates many genes involved in the transition G1/S and DNA synthesis (27). In addition to transcriptional repression, prohibitin can induce p53-mediated transcription, indicating that prohibitin may have dual functions in modulating transcription (28).

In a study conducted by Joshi et al. (29), the authors supported this theory by demonstrating that prohibitin can differentially regulate the Yin-Yang 1 and caspase 7 gene promoter activities. Additional functions related to prohibitin were linked to cell apoptosis (30). In this series, prohibitin was exclusively presented in Group 2, supporting its potential tumor suppressor activity. The critical functions of prohibitin in growth control and transcriptional regulation clearly indicate the need for further investigations to elucidate its importance in SCCP development.

To our knowledge this is the first study that analyzed penile tumors through proteomics technologies. Unfortunately, as all samples in our analysis were typed as HPV +, it was not possible to perform a comparison concerning HPV status in the group with tumor. As the selected patients were positive for HPV DNA, this fact can cause false negative for complement proteins. The variability could have been better analyzed if there were compared to patients with cancer of the penis, whose tests did not reveal the presence of HPV. The proteomic consequences of HPV infection in penile carcinoma are not known. Analysis of differentially expressed proteins by HPV status revealed enrichment of proteins involved in epithelial cell development, keratinization and extracellular matrix organization in HPV- oropharyngeal carcinoma (OPC), whereas enrichment of proteins in DNA initiation and replication and cell cycle control was found for HPV+ (OPC) (31). Due to the rarity of penile tumors and the high percentage of HPV positive in our samples (8, 13) it is difficult to compare the tumors according to HPV status. However, a second study is underway to compare our results and identify the presence or absence of complement in tissue of SCCP patients negative for HPV.

## CONCLUSIONS

We identified a large number of proteins in patients with penile cancer and in the control group. Some of these proteins, found in the first group, are also directly involved in the development of other types of cancers and therefore, suitable for analysis. Further studies are needed to corroborate these findings and to determine the usefulness of each discussed protein in the clinical scope of SCCP patients. Remarkably, this work reinforces that the C3 complement protein is a strong biomarker candidate for evaluating SCCP patients. Further studies should be conducted comparing samples positive for HPV with other HPV negative.
